# Amyloidosis of the tongue: a rare case report

**DOI:** 10.1016/j.bjorl.2023.101286

**Published:** 2023-06-28

**Authors:** Ying Tao, Xiaoling Qiu, Fan Ye, Zhencheng Liao, Pingan Wu

**Affiliations:** a The University of Hong Kong-Shenzhen Hospital, Department of Surgery, Division of Otolaryngology, Head and Neck Surgery, Shenzhen City, China; b The University of Hong Kong-Shenzhen Hospital, Department of Hematology, Shenzhen City, China

**Keywords:** Amyloidosis, Amyloidosis of the tongue, Maxillofacial surgery, AL

## Abstract

• This patient presented with clinical symptoms of tongue enlargement and stiffness.
• AL amyloidosis requires a pathological biopsy for diagnosis.
• Treatment involves chemotherapy and local surgical interventions.
• Misdiagnosis or delayed diagnosis of AL amyloidosis is common.

## Introduction

Amyloidosis of the tongue is a disease that involves systemic amyloidosis affecting the tongue tissue, which can cause protein metabolism disorders in the tongue tissue.^1^ The deposition of special insoluble amyloid proteins in the tongue tissue can manifest as widespread enlargement of the tongue, pain, nodules and papules on the tongue, and even bleeding and secondary infections.^2^ The patient's speech, swallowing, and breathing functions can be affected. In later stages, patients often die due to irreversible heart function decline caused by the deposition of amyloid proteins.^3^ However, systemic amyloidosis invading tongue tissue worldwide is extremely rare, and combined with atypical early symptoms, patients often mistake it for oral ulcers, missing the best diagnosis and treatment opportunities.^4^ Clinical doctors should be more aware of this disease and vigilant. This article reports a rare case of amyloidosis of the tongue. The patient's tongue began to gradually swell and repeatedly ulcerate four years ago, mistaking it for oral ulcers until it affected eating and speech, and was diagnosed with tongue amyloidosis by histopathology.

## Case report

**Figure 1:**
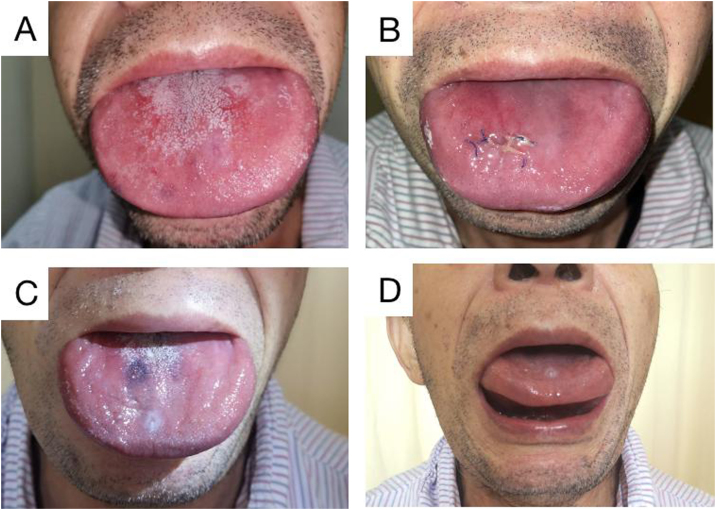
Physical examination of the tongue. Preoperative examination showed that the patient’s tongue was swollen, wider than the periodontal area, and the gum was pressed outward. The tongue is hard, and its activity is limited (A). Reswelling of the tongue was observed a week after surgery (B). 3 months after surgery (2 months after chemotherapy), the tongue was reduced (C). 3 months after surgery (2 months after chemotherapy), the tongue body can be lifted (D).

The patient in this case was a 71-year-old man who had been experiencing swelling and stiffness in his tongue for the past three years. He had also developed repeated ulcers that affected his ability to swallow, speak, and breathe. During the physical examination, it was noted that the patient's tongue was significantly enlarged and had tooth marks on the lingual margin. The tongue appeared to be of hard quality, with lip and buccal tilt of all teeth in the mouth. Loose teeth were also observed (Fig. 1A‒D).

**Figure 2:**
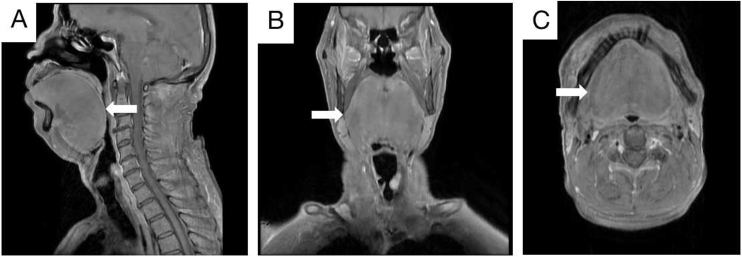
Enhanced MRI scan of nasopharynx and neck. The soft tissue of the tongue was obviously swollen, and the pharynx was compressed and narrow. No obvious signs of space occupation were observed. Sagittal position (A; White arrow). Coronal position (B; White arrow). Axial position (C; White arrow).

The patient had a previous history of hypertension and had smoked for many years. However, there was no special family history. An enhanced MRI of the nasopharynx revealed a mass on the left vocal cord and significant swelling of the soft tissue of the tongue (Fig. 2A‒C). Additionally, a chest CT showed multiple solid nodules in both lungs and thickening of the trachea and bronchus walls. Pulmonary ventilation function tests indicated severe mixed ventilation dysfunction, with FEV1 accounting for only 36.1% of the estimated value and FEV1/FVC accounting for 52.33%.

**Figure 3:**
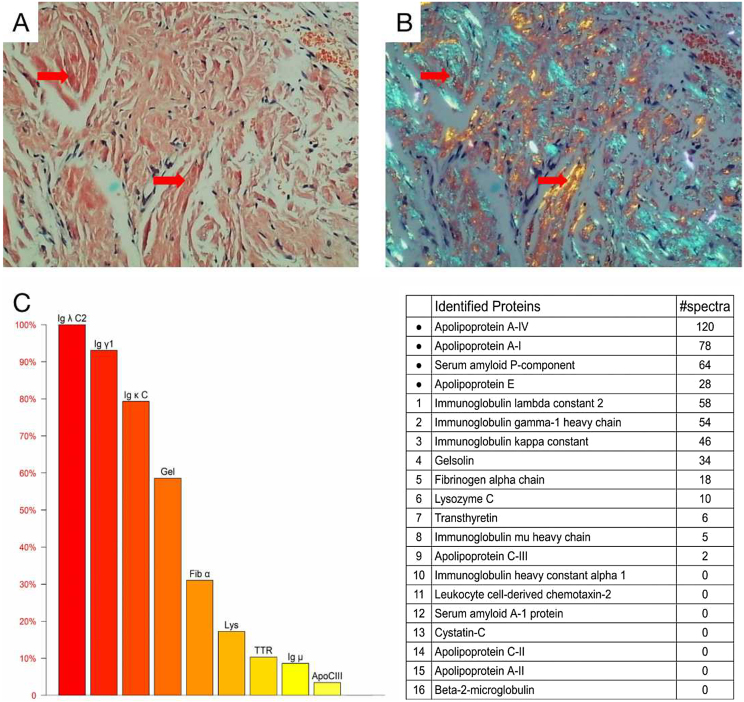
Histological and proteomic characteristics of AL amyloidosis. Tongue biopsy stained with Congo red revealed extensive amyloid deposition (A; Red arrow), the characteristic abnormal colors of orange and green were observed under a polarized light microscope (B; Red arrow). Proteomic analysis showed that high abundance of amyloid chaperone protein ApoAIV, SAP, ApoE was identified. The relative abundance of Igλ was the highest among the known proteins, suggesting that it was ALλ (C).

Cardiac color ultrasound and magnetic resonance imaging were consistent with invasive cardiomyopathy. A blood test showed a significantly increased serum free lambda light chain level (1135.52 mg/L) and a low serum free kappa/free lambda ratio of 0.0168. Bone marrow biopsy revealed 5.4% monoclonal plasma cells. The diagnosis of light chain (AL) amyloidosis was confirmed through a tongue biopsy, which showed apple green birefringence upon Congo red staining. Mass spectrum analysis indicated AL-Lambda type amyloidosis (Fig. 3A‒C).

Due to poor cardiopulmonary function, the patient was unable to undergo another surgery and was instead transferred to the Department of Hematology for bortezomib-based chemotherapy after multidisciplinary discussion. After two months of chemotherapy, the tongue body visibly reduced in size and could already complete lifting movements. However, the patient's cardiopulmonary function had not yet recovered, so chemotherapy continued.

## Discussion

Systemic immunoglobulin light chain (AL) amyloidosis is a rare disease where light chains (LCs) convert from soluble to fibrous aggregates in tissues leading to organ damage and dysfunction.^1^ Kyle et al.'s study reported that the annual incidence of AL amyloidosis was 8–10 cases per million population, with peak onset at ages 60–79, and most patients being male.^5^ Symptoms of AL amyloidosis are not usually recognized until the late stage, and studies show that nearly 40% of patients took over a year from symptom onset to diagnosis, with about 30% seeing at least five physicians before diagnosis.^4^ Typically, diagnosis of AL amyloidosis occurs during the terminal stage of the disease, and if cardiac involvement is present, the risk of death within a few months is high.^6^ Misdiagnosis and delayed diagnosis are significant issues with AL amyloidosis.

AL amyloidosis can affect multiple organs, including the heart (75%), kidneys (65%), soft tissues (15%), liver (15%), nervous system (10%), and gastrointestinal tract (5%).^7^ Cardiac involvement can lead to signs and symptoms of heart failure, while renal involvement may result in massive albuminuria, severe edema, and hypoalbuminemia. If the liver is affected, patients may experience liver enlargement and liver enzyme elevation, while nerve invasion can cause peripheral neuropathy. Skin and blood vessel involvement may lead to congestion and purpura.^8^

The disease's impact on survival is significant when multiple vital organs are involved, with a median survival time of only six months when both the heart and kidneys are affected.^7^ Involvement of the head and neck is less common, with the tongue being the most commonly affected area, accounting for 15%–20% of cases.^9^ Tongue involvement is characterized by slow, irreversible swelling, nodules, papules, plaques, and mucous plaques indicating large attachment.^2^ Early symptoms are often not obvious until lisp, dysphagia, or dyspnea occur. Amyloidosis is the most commonly reported cause of acquired tongue hyperplasia,^10^ but other conditions such as tuberculosis, lymphangioma, hypothyroidism, acromegaly, tongue stalk, sarcoidosis, muscular hyperplasia, and Beckwaite syndrome should also be considered as potential causes.^5^

The diagnosis of AL amyloidosis relies on a pathological biopsy of the affected tissue. Amyloid deposits appear as an extracellular eosinophilic amorphous substance under a light microscope in HE stained sections. However, other deposits such as collagen fibers, fibrin, other Ig deposits, or extracellular matrix may have a similar appearance. To address this issue, Congo red staining is utilized, which binds specifically to the amyloid β-sheet structure, resulting in brick-red staining and apple-green birefringence under polarized light.^11^ Additionally, determining the therapeutic protein is crucial for subsequent treatment. Immunohistochemical staining is a common method used to identify protein deposition. However, various factors such as serum contamination leading to high background, protein epitopes lost after formalin treatment, and lack of specific antibodies make it difficult to achieve high specificity and sensitivity. Currently, the development and application of proteomic analysis based on Mass Spectrometry (MS) is expected to resolve these issues.^12^

Treatment of AL amyloidosis involves a combination with chemotherapy to prevent production of amyloid light chain protein by targeting abnormal plasma cells and save organ function.^1^ Chemotherapy regimens based on retrospective studies and expert consensus, include MDex or CTDa; 65%–75% of patients receiving this program associated with therapeutic response in 3–4 months.^13^, ^14^ Bortezomib is a new class of drugs for treatment of myeloma, with high sensitivity in plasma cells of AL amyloidosis patients.^3^ Bortezomib combined with MDex superior to traditional regimens, showing more frequent and deeper hematologic responses and prolonged overall survival.^14^ Dara-CyBorD has become new standard-of-care treatment for AL amyloidosis, achieving deeper and lasting hematological response rate, and significant and high organ remission rates in Phase III clinical trial.^12^, ^15^

Patients with amyloidosis of the tongue experience difficulties in speech, swallowing, breathing, and even suffocation. Local surgical treatment may be helpful in reducing tongue hypertrophy and improving function. Traditional surgery is difficult and postoperative care is challenging, but plasma Radiofrequency Ablation (RFA) is a minimally invasive surgical technique that has shown great advantages in reducing tissue volume, protecting surface mucosa, and producing hemostatic effects with limited damage to surrounding tissue.^12^, ^16^, ^17^ The patient in this case was unable to tolerate general anesthesia due to poor cardiopulmonary function and did not undergo tongue volume reduction treatment. Systemic amyloidosis invading tongue tissue is rare and can be misdiagnosed or missed clinically, highlighting the need for increased awareness and multidisciplinary joint diagnosis and treatment when necessary.

## Conclusions

In summary, we present a rare case of tongue amyloidosis where the patient experienced tongue hypertrophy for years. The diagnosis was confirmed through a tongue biopsy, and after multidisciplinary discussion, bortezomib-based chemotherapy was chosen as the first course of treatment. Once cardiopulmonary function improves enough to allow for surgical intervention, we plan to perform RFA to decrease tongue size, thereby reducing the risk of airway obstruction and improving quality of life.

## Authors’ contributions

Ying Tao, Fan Ye, and Xiaoling Qiu wrote the first draft. Fan Ye, Xiaoling Qiu, and Pingan Wu contributed to patient evaluation and data collection. Ying Tao prepared the images. Fan Ye, Zhenhen Liao, and Pingan Wu administered the study and critically revised the manuscript. All authors contributed to the article and approved the submitted version.

## Funding

This study was supported by Natural Science Foundation of Shenzhen City (Grant Number: JCYJ20220530142413031).

## Ethics statement

The studies involving human participants were reviewed and approved by the Ethics Committee of the University of Hong Kong Shenzhen Hospital. The patients/participants provided their written informed consent to participate in this study.

## Conflicts ofinterest

The authors declare that the research was conducted in the absence of any commercial or financial relationships that could be construed as a potential conflict of interest.

## References

[1] Wechalekar A.D., Gillmore J.D., Hawkins P.N. (2016). Systemic amyloidosis. Lancet..

[2] Indu S., Roy I.D., Tewari R., Pramanik S. (2021). Oral amyloidosis: a case report and diagnostic algorithm. J Oral Maxillofac Pathol..

[3] Palladini G., Milani P., Malavasi F., Merlini G. (2021). Daratumumab in the treatment of light-chain (AL) amyloidosis. Cells..

[4] Lousada I., Comenzo R.L., Landau H., Guthrie S., Merlini G. (2015). Light chain amyloidosis: patient experience survey from the amyloidosis research consortium. Adv Ther.

[5] Matsuo F.S., Barbosa de Paulo L.F., Servato J.P., de Faria P.R., Cardoso S.V., Loyola A.M. (2016). Involvement of oral tissues by AL amyloidosis: a literature review and report of eight new cases. Clin Oral Investig..

[6] Ravichandran S., Lachmann H.J., Wechalekar A.D. (2020). Epidemiologic and survival trends in amyloidosis, 1987–2019. N Engl J Med..

[7] Palladini G., Merlini G. (2016). What is new in diagnosis and management of light chain amyloidosis?. Blood..

[8] Taylor N.A., Lugo-Somolinos A., Sayed C.J. (2016). Bruising and hemorrhagic vesicles on the tongue. JAMA Dermatol..

[9] Salman R., Lateef M., Iqbal I., Rehman A., Ul Islam M. (2011). Laryngeal amyloidosis: a case report. Indian J Otolaryngol Head Neck Surg..

[10] Dietrich E., Grimaux X., Martin L., Samimi M. (2022). Etiological diagnosis of macroglossia: systematic review and diagnostic algorithm. Ann Dermatol Venereol..

[11] Perfetto F., Moggi-Pignone A., Livi R., Tempestini A., Bergesio F., Matucci-Cerinic M. (2010). Systemic amyloidosis: a challenge for the rheumatologist. Nat Rev Rheumatol..

[12] Cohen A.D., Comenzo R.L. (2010). Systemic light-chain amyloidosis: advances in diagnosis, prognosis, and therapy. Hematol Am Soc Hematol Educ Program..

[13] Kastritis E., Dialoupi I., Gavriatopoulou M., Roussou M., Kanellias N., Fotiou D. (2019). Primary treatment of light-chain amyloidosis with bortezomib, lenalidomide, and dexamethasone. Blood Adv..

[14] Kastritis E., Wechalekar A.D., Dimopoulos M.A., Merlini G., Hawkins P.N., Perfetti V. (2010). Bortezomib with or without dexamethasone in primary systemic (light chain) amyloidosis. J Clin Oncol..

[15] Kastritis E., Palladini G., Minnema M.C., Wechalekar A.D., Jaccard A., Lee H.C., ANDROMEDA Trial Investigators (2021). Daratumumab-based treatment for immunoglobulin light-chain amyloidosis. N Engl J Med.

[16] Bucci T., Bucci E., Rullan A.M., Bucci P., Nuzzolo P. (2014). Localized amyloidosis of the upper gingiva: a case report. J Med Case Rep..

[17] Cui S., Cheng C., Tong Y. (2021). Experience of low-temperature plasma radiofrequency treatment of 53 patients with tongue hemangioma. Am J Otolaryngol..

